# Design and performance of a real-time RT-PCR assay for detection of influenza C viruses

**DOI:** 10.1016/j.jcv.2025.105874

**Published:** 2025-09-25

**Authors:** Bo Shu, William G. Davis, Ji Liu, Beth K. Thielen, Sarah Bistodeau, Brian Lynch, Christine M. Warnes, Jimma Liddell, Anna K. Strain, Jaime Christensen, Phili Wong, Natasha Burnett, Todd C. Davis, Marie K. Kirby

**Affiliations:** a National Center for Immunization and Respiratory Diseases, Centers for Disease Control and Prevention, Atlanta, United States; b Division of Pediatric Infectious Diseases, University of Minnesota Medical School, Minneapolis, United States; c Minnesota Department of Health, St. Paul, United States

**Keywords:** Influenza C virus, Diagnostic assay, Real-time RT-PCR

## Abstract

Influenza C virus (ICV) usually causes a mild upper respiratory tract infection in children and those infected are frequently co-infected with other respiratory viruses. However, there have only been a few hundred documented cases of ICV infection in humans as of the end of 2024. To better understand the epidemiology and clinical impact of ICVs, we developed an influenza C real-time RT-PCR (InfC rRT-PCR) assay that targets a highly conserved region of the matrix gene segment of ICVs. The analytical sensitivity evaluation demonstrated that the InfC rRT-PCR assay was highly sensitive, as it was able to detect as few as five RNA copies per PCR reaction and had robust reactivity over a range of viral RNAs from historical and recent ICVs. The analytical specificity evaluation confirmed the assay did not cross-react with any influenza A or B viruses tested, including several animal-origin viruses, or other common non-influenza respiratory viruses. The performance evaluation on clinical specimens demonstrated the assay was highly sensitive and specific for the detection of ICVs.

## 1. Introduction

Influenza C virus (ICV) was first identified in the United States (U.S.) in 1947 with humans being the primary host species and periodic detections in pigs, dogs and cattle [1-4]. ICVs more commonly infect children, often causing mild febrile upper respiratory tract infections; however, more severe symptoms, such as bronchitis and pneumonia, can occasionally occur due to lower respiratory tract infections [5,6]. ICV is often detected as co-infections with other respiratory viruses, including influenza A and B viruses (IAVs and IBVs) [7].

The ICV genome, which has seven segments, encodes a hemagglutinin-esterase fusion (HEF) protein that performs the functions of both the hemagglutinin and neuraminidase proteins of IAVs and IBVs. Based on HEF genetic and antigenic variance, ICVs are divided into six lineages, namely C/Taylor/1233/1947 (Taylor47), C/Kanagawa/1/76 (KA176), C/Yamagata/26/81 (YA2681), C/Aichi/1/81(AI181), C/Sao Paulo/378/82 (SP82), and C/Mississippi/80(MS80) [8,9].

Serosurveillance studies reveal a worldwide distribution pattern of ICVs; [5,10] however, relatively little is known about the epidemiology and clinical impact, with only a few hundred documented cases of ICV infections in humans as of the end of 2024. There are now several real-time RT-PCR (rRT-PCR) or conventional RT-PCR assays for the detection of ICV [11,12], but there are only a few reports on their use in ICV surveillance. To support several ICV investigations and surveillance programs nationally or internationally and enhance our understanding of the epidemiology and clinical impact of ICVs, we have developed an influenza C rRT-PCR (InfC rRT-PCR) assay that will sensitively and specifically detect ICVs.

## 2. Materials and methods

### 2.1. Influenza viruses and RNA extraction

ICVs were cultured in Madin-Darby Canine Kidney (MDCK) cells [13]. The ICVs used to evaluate analytical performance included three historical ICV strains—C/Aomori/1974 (KA176 lineage), C/Kansas/2/1979 (AI181 lineage) and C/Yamagata/10/1981 (Taylor/47 lineage) —and three recent ICV strains— C/Minnesota/29/2015 (SP82 lineage), C/Minnesota/05/2014 (KA176 lineage) and C/Minnesota/1/2016 (KA176 lineage).

IAV and IBVs used in this study were cultured in either MDCK cells or embryonated chicken eggs (ECE) [14]. The IAV and IBV virus titers were measured by tissue culture infectious dose-50 % (TCID_50_/ml) or egg infectious dose-50 % (EID_50_/ml) [15].

Viral RNA was extracted from 100 μL of virus isolates or clinical specimens and eluted in 100 μL elution buffer using the MagNA Pure Compact RNA isolation kit on a MagNA Pure Compact instrument (Roche Applied Science) according to manufacturer’s instructions. While the MagNA Pure Compact was recently sunset from manufacturing, we have shown comparable performance between this instrument and other similar extractors, such as the Qiagen EZ1 (data not shown).

### 2.2. InfC rRT-PCR assay primers/probes

We designed the InfC rRT-PCR assay based on nucleotide sequence data available from the National Center for Biological Information (NCBI) GenBank database and the Global Initiative on Sharing All Influenza Data (GISAID). The oligonucleotide primers and probe were designed to target a highly conserved region of the matrix (M) gene to allow universal detection of ICVs (Table 1). Specifically, we used PrimerExpress 3.0.1 software (Applied Biosystems, Foster City, USA) to design the primers/probes to have annealing temperatures of approximately 60 °C and 70 °C, respectively. We confirmed primer/probe specificity through comparisons with sequence data from NCBI and GISAID databases.

We evaluated two quencher chemistries for Taqman^®^ hydrolysis probes, namely, Blackhole Quencher^™^ (BHQ^™^) and the ZEN^™^ Double-Quenched Probe. Both probe types were labeled at the 5′-end with the reporter molecule, 6-carboxyfluorescein (FAM). The BHQ^™^ probe contained a Black Hole Quencher 1 (BHQ-1) at the modified “T” residue, and a 3’-end Spacer 6 (Sp6, Biosearch Technologies, Inc., Novato, CA) (Table 1). Meanwhile, the ZEN^™^ Double-Quenched Probe contained a ZEN Quencher between base residues 9 and 10, and an Iowa Black FQ quencher at the 3′-end (Integrated DNA Technologies, Coralville, IA). The CDC Biotechnology Core Facility (CDC, Atlanta, GA) synthesized all primers and dual-labeled Taqman^®^ hydrolysis probes.

A modified probe was also designed with a degenerate base “Y” at nucleotide position 9.

### 2.3. rRT-PCR reaction conditions

We optimized reaction parameters for the InfC rRT-PCR assay using the Invitrogen SuperScript^™^III Platinum^®^ One-Step quantitative RT-PCR Kits (Life Technologies) on the Applied Biosystems^™^ (AB) 7500 Fast Dx Real-Time PCR systems. A thermal gradient analysis of InfC rRT-PCR assay reactions indicated comparable performance at annealing temperature range from 50 °C to 62.5 °C (Table S1, Figure S1). Consequently, we set the final reaction annealing temperature at 55 °C (Table S1, Figure S1).

Reaction conditions for rRT-PCR were based upon U.S. Food and Drug Administration (FDA)–approved CDC Human Influenza Virus Real-Time RT-PCR Detection and Characterization Panel (CDC Flu rRT-PCR Dx Panel): Total reaction volume for the rRT-PCR reactions performed was 25 μl, with primer and probe reaction concentrations at 0.8 μM and 0.2 μM, respectively. The thermocycling rRT-PCR conditions were as follows: 50 °C for 30 min, *Taq* activation at 95 °C for 2 min and 45 cycles of 95 °C for 15 s and 55 °C for 30 s [16,17].

### 2.4. Analytical sensitivity and specificity

The analytical sensitivity and specificity of the InfC rRT-PCR assay were evaluated by using the ZEN^™^ probe.

Analytical sensitivity of the InfC assay was determined on 3 historical strains, 3 recent strains, and a synthetic RNA sequence derived from the M gene of C/Minnesota/01/2016 (KA176 lineage) virus (Armored RNA Quant^®^ CDC-9, AsuraGen, Inc.). The analytical specificity was evaluated using 14 IAVs, 6 IBVs, and 15 non-influenza viruses that commonly infect the human respiratory tract via the nasopharynx.

### 2.5. Clinical performance of InfC rRT-PCR assay

Since 2008, we have supported a number of ICV detection and surveillance programs, including one with the Minnesota Department of Health. In 2017, we received 39 specimens collected from Minnesota between December 2014 and February 2016 that tested positive for InfC virus using the CDC InfC rRT-PCR assay. Of these, 32 specimens were collected from patients who were admitted with severe acute respiratory illness (ARI), while seven were from outpatients with influenza-like illness (ILI) or ARI [18].

We tested the 39 suspect ICV specimens, which were all collected from the upper or lower respiratory tract of patients, along with 30 additional respiratory specimens from unrelated patients received by CDC for testing using the InfC rRT-PCR assay (ZEN^™^ probe) and the CDC Flu rRT-PCR Dx Panel-Influenza A/B Typing Kit. We further sequenced the HEF genes of ICV-positive samples according to CDC’s protocol to confirm the rRT-PCR result [18].

## 3. Results

### 3.1. Establishing the influenza C rRT-PCR assay

The InfC rRT-PCR assay was developed using the ABI 7500 Fast Dx Real-time PCR system for the qualitative detection and characterization of ICV RNA in respiratory specimens from patients presenting with ILI and/or ARI. Based on the thermal gradient analysis, we set the annealing temperature at 55 °C, which is identical to current IAV and IBV diagnostic assays of the CDC Flu rRT-PCR Dx Panel [16,17] and accommodates potential nucleotide mismatches in the primer/probe regions due to virus genetic variability.

**Fig. 1.**
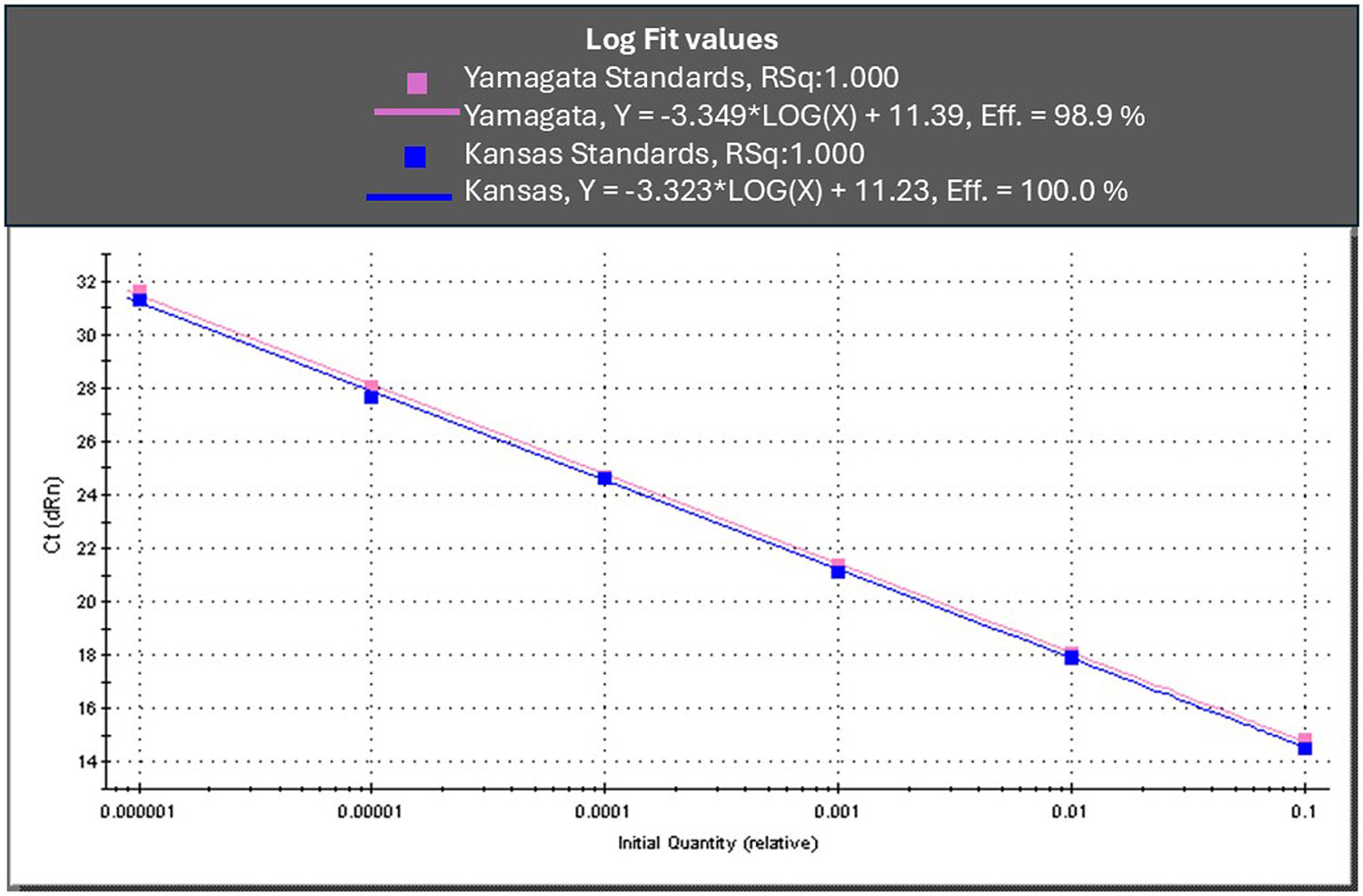
Reaction efficiency of InfC real-time RT-PCR assay. Reaction efficiencies/RSqs were determined by testing ten-fold serial dilutions of viral RNA of C/Yamagata/10/1981(pink) and C/Kansas/2/1979(blue), respectively.

We measured reaction efficiencies of the InfC assay by testing ten-fold dilution series of viral RNA extracted from two historical ICV strains, C/Kansas/2/1979 (AI181 lineage) and C/Yamagata/10/1981 (YA2681 lineage). Overall, we observed 98.9–100 % efficiency (Fig. 1). Furthermore, both hydrolysis probe types— ZEN^™^ and BHQ^™^ — yielded comparable Ct values for C/Minnesota/29/2015 (SP82 lineage) tested using both quencher chemistries (Table 2)

### 3.2. Analytical sensitivity

**Fig. 2.**
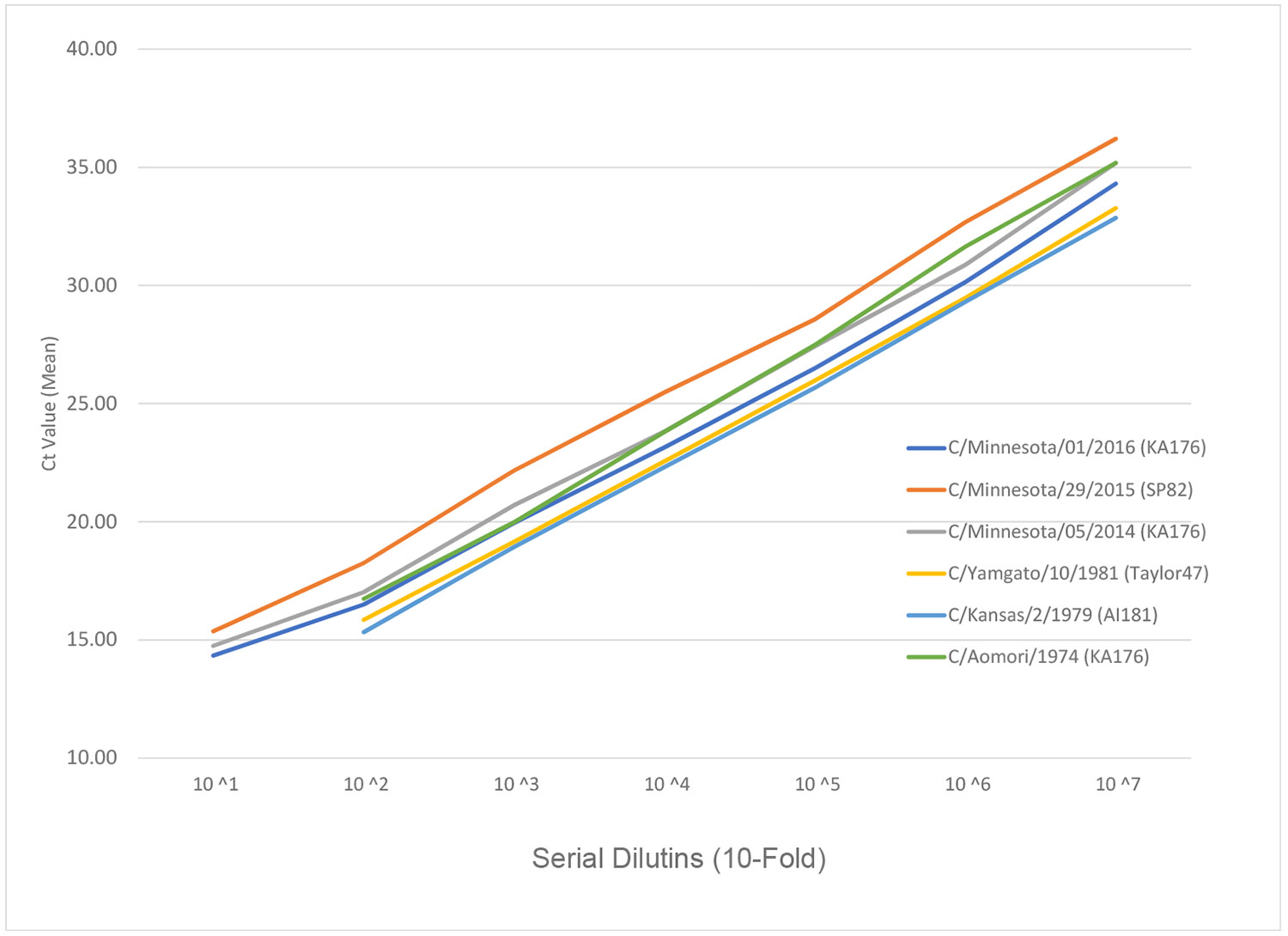
Analytical sensitivity evaluation with historical and recent influenza C viruses (test in triplicates).

To determine the analytical sensitivity of the ICV assay, we extracted viral RNA from three historical ICV strains—C/Aomori/1974 (KA176 lineage), C/Yamagata/10/1981 (Taylor47 lineage) and C/Kansas/2/1979 (AI181 lineage)—and three recent ICV strains—C/Minnesota/29/2015 (SP82 lineage), C/Minnesota/05/2014 (KA176 lineage) and C/ Minnesota/1/2016 (KA176 lineage)—then tested a 10-fold dilution series according to the optimized RT-PCR parameters. All six viral RNAs tested positive for ICV by the InfC rRT-PCR assay, even at 10^−7^ dilution (Fig. 2 and Table S2).

We also tested a synthetic RNA derived from ICV, C/Minnesota/1/2026(KA176 lineage) (Armored RNA Quant^®^ CDC-9, AsuraGen, Inc.) to determine the minimum RNA copy number which the InfC rRT-PCR assay could consistently detect. Overall, we determined the limit of detection (LoD) at 5 copies of RNA per reaction (Table 3).

### 3.3. Analytical specificity

To demonstrate that the ICV assay retained exclusivity of IAVs, IBVs, and other common respiratory pathogens, we tested several of these pathogens on the InfC assay to confirm lack of positivity. In total, we tested six each of seasonal IAV or IBV subtypes or lineages, including the 2009 pandemic influenza A(H1N1) (H1N1pdm09) and influenza A (H3N2) subtypes. We also tested two avian influenza viruses; one each of A(H5N1) and A(H7N9) viruses (Table 4). All IAVs and IBVs tested negative by the InfC rRT-PCR assay. Likewise, all 15 non-influenza viral pathogens also tested negative (Table S3).

### 3.4. rRT-PCR detection of Influenza C viruses from human specimens

Thirty-nine ICV suspected specimens that had tested positive with the CDC InfC rRT-PCR assay from the Minnesota Department of Health were subjected to a confirmatory InfC rRT-PCR test in 2017 by the CDC’s Influenza Division. All 39 specimens also tested positive with the InfC rRT-PCR assay performed at CDC. The specimens were also tested with the CDC Flu rRT-PCR Dx Panel-Influenza A/B Subtyping Kit. All specimens tested positive for the Human Rnase P (RP) assay indicating the specimens were of good quality. Thirty-eight of the 39 specimens tested negative by the universal-influenza A and B (InfA and InfB) assays, and one specimen tested positive for InfA (Table 5).

We also evaluated the clinical performance of the InfC rRT-PCR assay by testing an additional 30 residual clinical respiratory specimens previously tested for influenza, including 10 specimens positive for IAVs, 10 specimens positive for IBVs and 10 influenza negative specimens (Non influenza); the results were in 100 % agreement with the expected result for each specimen (Table 5).

The HEF gene genetic sequence analysis provided additional confirmation of the InfC rRT-PCR positive result. Thirteen of the 39 HEF genes from the positive samples were SP82 lineage, while 26 were KA176 lineage.

### 3.5. In silico analysis of primers and probes of InfC assay

To assess potential mismatches between ICV M gene sequences and the primers and probes designed, we performed *in silico* analysis that compared the primers and probe against the ICV M gene sequences that had been deposited in GISAID between 2000 and 2024. Within the forward primer region, we found only one virus with a mismatch. Within the reverse primer region, 6.3 % (21/332) and 1.8 %(6/332) viruses had one or two mismatches, respectively. Within the probe region, 29.0 % (98/338) viruses had one mismatch and 3.6 % viruses (12/338) had two mismatches. Additionally, we analyzed the probe region sequences for a cytosine to thymine change at nucleotide position 9; in total, 24.0 % (81/338) viruses contained this change, including 9 viruses with two mismatches. As a result, within the modified probe, 6.8 % (26/338) and 0.9 (3/338) viruses had only one or two mismatches, respectively (Table 6).

## 4. Discussion

The InfC assay presented here is intended for qualitative detection of ICV using rRT-PCR technology. The analytical sensitivity of the InfC assay demonstrated its high performance. Herein, we show that the assay has robust reactivity across a dynamic range of quantified synthetic RNA copy numbers, and is highly sensitive, detecting as few as 5 viral RNA copies per PCR reaction. Importantly, our data indicated that the InfC rRT-PCR assay was specific to ICVs, as we observed no cross-reactivity with other viruses tested in our exclusivity evaluation, including high-titer concentrations of seasonal IAV or IBV subtypes or lineages, as well as A(H5N1) and A(H7N9) viruses and other common respiratory viruses. The performance evaluation of clinical specimens further confirmed the assay’s high sensitivity and specificity for detecting ICVs. Additionally, we found that the assay functions equivalently well with both BHQ and ZEN fluorescent hydrolysis probe quencher chemistries.

Influenza viruses rapidly adapt and evolve, generating diversity through mutations and genetic reassortment [19,20]. This process can also impact the accuracy of diagnostic testing; making it imperative to monitor viral genetic sequences for potential mismatches that may affect assay performance. The InfC rRT-PCR primers and probes were designed based on publicly available sequences from 1947 to 2007. To assess the compatibility of our primers and probes with both historical and recent virus strains, we conducted *in silico* analysis on 366 ICV M gene sequences deposited in GISAID between 2000 and 2024. The results of these analyses showed a low mismatch rate, with fewer than 10% of viruses exhibiting mismatches within the primer region. Only 3.6% of viruses had two mismatches in the probe region, with the majority of mismatches occurring at the C9T position in the probe. We evaluated the modified probe by introducing an ambiguous base Y (C+T) at nucleotide position nine of the probe, and our assay retained comparable sensitivity even among the virus containing a mismatch (Table 2). Nonetheless, the probe can be updated to the modified probe sequence to reduce the number and rate of mismatches. Based on this analysis, we concluded that the primer/probe region of our InfC RT-PCR assay is highly conserved, allowing for the detection of both historical and recent ICV strains.

The use of the InfC assay coupled with the CDC Flu rRT-PCR Dx panel kits serves as an effective method for detection and/or surveillance of influenza A, B, or C viruses. This assay will help to support ICV investigations and surveillance projects across the U.S. and other countries. Current projects, including Vaccine Effectiveness (VE) studies, the National Vaccine Surveillance Network (NVSN), the Influenza Incidence Surveillance Project (IISP), and the TaqMan Array Card project [18,21,22] will benefit by having the ability to detect rare InfC virus infections. Suspect InfC positive specimens from collaborators, including state and local public health laboratories, can also be confirmed with this test. These results will lead to additional efforts to sequence the HEF gene or whole genome of influenza C viruses to expand the availability of these data in public databases and to better assess the global distribution of ICV genetic lineages. Collectively, these projects aim to determine the burden of ICV infection in children, including those detected through ARI in-patient testing and emergency department visits. Additionally, the assay will help to provide the necessary testing to better describe the epidemiologic, demographic and clinical features of ICV in children and the population at large. As of today, the CDC’s InfC rRT-PCR assay has detected approximately 200 specimens of influenza C virus infection, a subset of which have been confirmed through sequencing [18,21,22].

## Supplementary Material

Supplementary Material

## Figures and Tables

**Table 1.** Primers and probes used in the influenza C real-time RT-PCR assay

Primer/Probe		Sequence 5'-3'	Nucleotide Position^1^
Forward Primer		AGA TGG GAG AGA TGG TGT GGA GA	939-961
Probe	ZEN^™^ probe	ATT GCC CTC <ZEN> TCT AGG GAG AGA CTT GAC CTG^2^	1008-1037
	BHQ^™^ probe	ATT GCC CTC TC"T" AGG GAG AGA CTT GAC CTG ^3^	
	Modified probe	ATT GCC CTY TC”T” AGG GAG AGA CTT GAC CTG^3^	
Reverse primer		TTG GTG AGT TGT CGG TTT CGT	1074-1054

1 Nucleotide Positions are according to M gene of C/Minnesota/1/2016 (CY239485)

2 Probe labeled at the 5'-end with the reporter 6-carboxyfluorescein (FAM), a ZEN Quencher between base residues 9 and 10, and an Iowa Black FQ (IBFQ, IWBkFQ) quencher at the 3'-end (Integrated DNA Technologies, Coralville, IA)

3 Probe labeled at the 5'-end with the reporter molecule 6-carboxyfluorescein (FAM), a Black Hole Quencher 1 (BHQ-1) at the modified “T” residue, and a 3’-end Spacer 6 (Sp6) (Biosearch Technologies, Inc., Novato, CA)

**Table 2.** Performance evaluation of InfC real-time RT-PCR with three probes (tested in triplicates)

Influenza C viruses	Ct values of InfC rRT-PCR (Mean±SD)		
C/Minnesota/29/2015_SP82 lineage*	ZEN^™^ probe	BHQ^™^ probe	Modified probe
10 ^−1^	15.37±0.05	15.53±0.06	15.41±0.03
10 ^−2^	18.25±0.10	18.76±0.07	18.57±0.09
10 ^−3^	22.16±0.07	22.09±0.19	22.01±0.14
10 ^−4^	25.48±0.34	25.54±0.17	25.21±0.13
10 ^−5^	28.57±0.43	28.89±0.14	28.75±0.35
10 ^−6^	32.68±0.63	32.95±0.24	32.37±0.09
10 ^−7^	36.21±0.99	36.97±0.35	36.48±0.29

* C9T mimatch with the InfC assay probe

**Table 3.** Assay limit of detection determined using quantified synthetic RNA ^a^ (tested in triplicates)

No. of RNA copies/reaction	Ct values of InfC rRT-PCR		
50,000	23.04	23.25	23.59
5000	26.12	27.01	27.04
500	30.46	30.75	29.95
50	33.16	33.61	34.07
5	36.23	36.81	36.36

a The RNA material [Armored RNA Quant^®^ CDC-9, received from AsuraGen, Inc] includes M gene region sequences derived from C/Minnesota/1/2016 (KA176 lineage) virus.

**Table 4.** Analytical specificity (exclusivity) testing with influenza A or B viruses

Influenza A or B viruses	Subtype or lineage	Infectious titer (ID_50_/ml)	Result of rRT-PCR	
			InfA/InfB	InfC
A/Beijing/262/1995	A(H1N1)	10^6.0a^	+/−	-
A/New Caledonia/20/1999	A(H1N1)	10^6.6b^	+/−	-
A/Solomon Islands/03/2006	A(H1N1)	10^6.2a^	+/−	-
A/Brisbane/59/2007	A(H1N1)	10^8.4b^	+/−	-
A/California/07/2009	A(H1N1) pdm09	10^7.5b^	+/−	-
A/Michigan/45/2015	A(H1N1) pdm09	10^7.2b^	+/−	-
A/New York/55/2004	A(H3N2)	10^6.4b^	+/−	-
A/Wisconsin/67/2005	A(H3N2)	10^6.5b^	+/−	-
A/Hawaii/08/2006	A(H3N2)	10^7.8a^	+/−	-
A/Uruguay/716/2007	A(H3N2)	10^8.2b^	+/−	-
A/Afghanistan/2903/2008	A(H3N2)	10^5.0a^	+/−	-
A/Texas/50/2012	A(H3N2)	10^6.2a^	+/−	-
A/Vietnam/1203/2014	A(H5N1)	10^6.8a^	+/−	-
A/Anhui/1/2013	A(H7N9)	10^8.9a^	+/−	-
B/Beijing/184/1993	B/YAM	10^5.0a^	−/+	-
B/Florida/07/2004	B/YAM	10^6.0b^	−/+	-
B/Victoria/304/2006	B/VIC	10^8.2b^	−/+	-
B/Brisbane/03/2007	B/YAM	10^8.4b^	−/+	-
B/Brisbane/60/2008	B/VIC	10^6.9b^	−/+	-
B/Phuket/3073.2013	B/YAM	10^7.9b^	−/+	-

a TCID50/ml;

b EID50/ml

**Table 5.** Clinical performance of InfC real-time RT-PCR assay and the CDC influenza A/B Typing Kit assays

Specimen type	Number	Result of rRT-PCR			
		InfC	InfA	InfB	Rp
Influenza C	39	39*	1	0	39
Influenza A	10	0	10	0	10
Influenza B	10	0	0	10	10
Non influenza	10	0	0	0	10

InfC assay positive result was confirmed by HEF genetic sequence analysis. thirteen of the 39 HEF genes are from the SP82 lineage, while 26 are from the KA176 lineage.

**Table 6:** *In silico* analysis of primer probes of influenza C rRT-PCR assay

		Number (%) of mismatched nucleotides				Total
		0	1	2	≥3	
Number of analyzed sequences(%)	Forward primer	365(99.70)	1 (0.30)	0(0.0)	0(0.0)	366
	Reverse Primer	305(91.87)	21(6.33)	6(1.81)	0(0.0)	332
	Probe	228(67.46)	98(29.00)	12(3.55)	0(0.0)	338
	Modified probe	312(92.31)	23(6.80)	3(0.89)	0(0.0)	

The M gene sequences deposited in GISAID between 2000-2014 were analyzed

## Appendix A. Supporting information

Supplementary data associated with this article can be found in the online version at doi:10.1016/j.jcv.2025.105874.
