# Supplementary material for: Design and performance of a real-time RT-PCR assay for detection of influenza C viruses
Source: J Clin Virol. Author manuscript; Available in PMC 2026 Mar 10. (PMC12974223; doi:10.1016/j.jcv.2025.105874)
Supplement: Supplementary Material [file NIHMS2147245-supplement-Supplementary_Material.docx]

Table S1. Thermal gradient analysis of InfC real-time RT-PCR assay

| Annealing Tm (°C) | Ct values of InfC rRT-PCR (Mean±SD) |
| --- | --- |
| 50.0 | 28.45±0.05 |
| 51.0 | 28.25±0.04 |
| 53.0 | 28.57±0.14 |
| 55.9 | 28.32±0.10 |
| 59.5 | 28.24±0.10 |
| 62.5 | 28.12±0.08 |
| 64.1 | 28.12±0.02 |
| 65.0 | 28.21±0.02 |

Thermal gradient analysis was performed using Invitrogen reagent with viral RNAs from C/Yamagata/11/1981(Taylor47 lineage) on a Bio-Rad CFX96™ Real-Time PCR Detection System.

Table S2. Analytical sensitivity evaluation with historical and recent influenza C viruses (test in triplicate)

Table S3. Analytical specificity (exclusivity) with common respiratory viruses

| Strain | Respiratory Virus | Titer | Result of InfC rRT-PCR |
| --- | --- | --- | --- |
| Echo 6 | Enterovirus | 10^6.9a^ | - |
| Ad. 71 | Human Adenovirus, type 1 | 10^9.2a^ | - |
| S-1058 | Human Adenovirus, type 7a | 10^7.1a^ | - |
| OC43 | Human Coronavirus | 50.4^b^ | - |
| 229E | Human Coronavirus | 31.6^b^ | - |
| 1A | Human Rhinovirus A | 10^5.8a^ | - |
|  | Human Parainfluenza 1 virus | 3.0^b^ | - |
| Greer | Human Parainfluenza 2 virus | 10^3.1a^ | - |
| C-243 | Human Parainfluenza 3 virus | 10^7.9a^ | - |
| CH93-18b | Respiratory Syncytial Virus (RSV) | 10^6.8a^ | - |
| KOS | Herpes Simplex Virus | 10^8.4a^ | - |
| AV92-3 | Varicella-zoster Virus | 10^4.4a^ | - |
| B95-8 | Epstein Barr Virus | 1.7^b^ | - |
| Edmonston | Measles Virus | 10^5.2a^ | - |
| AD-169 | Cytomegalovirus | 10^6.9a^ | - |

^a^TCID50/mL ; ^b^ng/mL •IFU/mL

Figure S1**.** Amplification chart of thermal gradient analysis of InfC real-time RT-PCR assay
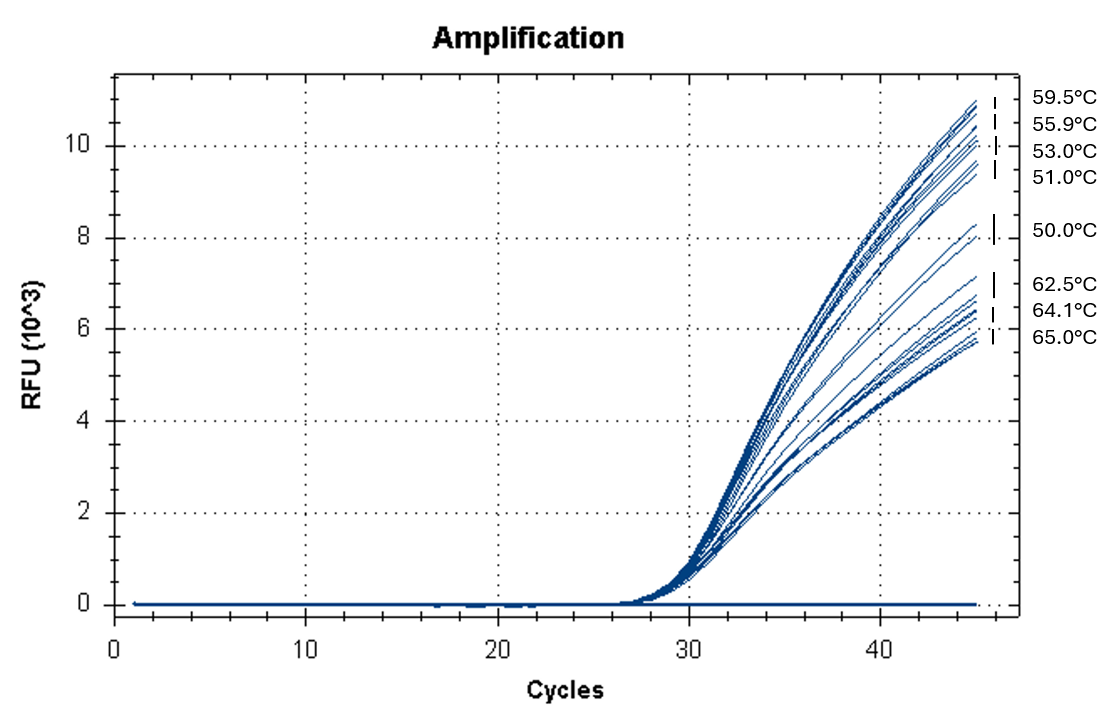
Thermal gradient analysis was performed using viral RNA of C/Yamagata/11/1981 (Taylor47 lineage) with annealing temperatures ranging from 50.0-65.0°C.
